# Collaborative care model for diabetes in primary care settings in Qatar: a qualitative exploration among healthcare professionals and patients who experienced the service

**DOI:** 10.1186/s12913-021-06183-z

**Published:** 2021-03-02

**Authors:** Sara Abdulrhim, Sowndramalingam Sankaralingam, Mohamed Izham Mohamed Ibrahim, Mohammed Issam Diab, Mohamed Abdelazim Mohamed Hussain, Hend Al Raey, Mohammed Thahir Ismail, Ahmed Awaisu

**Affiliations:** 1 Department of Clinical Pharmacy and Practice, College of Pharmacy, QU Health, Qatar University, Doha, Qatar; 2 Qatar Petroleum Healthcare Center, Dukhan, Qatar

**Keywords:** Diabetes, Primary healthcare, Collaborative care, Patient perspective, Healthcare professional perspective

## Abstract

**Background:**

Diabetes mellitus is highly prevalent and associated with huge economic burden globally. The conventional care and management of diabetes mellitus is highly fragmented and complex, warranting the need for a comprehensive Collaborative Care Model (CCM). Little is known about the perception of patients with diabetes and their healthcare providers about CCM, its barriers and facilitators. This study aimed to explore the value of CCM in diabetes care at a primary healthcare (PHC) setting from the perspective of patients with diabetes and healthcare professionals (HCPs), in an effort to expand our current knowledge on collaborative care in diabetes at primary care level for the purpose of quality improvement and service expansion.

**Methods:**

Using an exploratory case study approach, semi-structured interviews were conducted among patients and HCPs who encountered CCM in Qatar during 2019 and 2020. The semi-structured interviews were transcribed verbatim and the data were analysed and interpreted using a deductive-inductive thematic analysis approach.

**Results:**

Twelve patients and 12 HCPs at a diabetes clinic participated in one-to-one interviews. The interviews resulted in five different themes: the process and components of collaborative care model (four subthemes), current organizational support and resources (three subthemes), impact of collaborative care model on diabetes outcomes (three subthemes), enablers of collaborative care model (three subthemes), and barriers to collaborative care model (three subthemes). The participants indicated easy access to and communication with competent and pleasant HCPs. The patients appreciated the extra time spent with HCPs, frequent follow-up visits, and health education, which empowered them to self-manage diabetes. HCPs believed that successful CCM provision relied on their interest and commitment to care for patients with diabetes. Generally, participants identified barriers and facilitators that are related to patients, HCPs, and healthcare system.

**Conclusions:**

The providers and users of CCM had an overall positive perception and appreciation of this model in PHC settings. Barriers to CCM such as undesirable attributes of HCPs and patients, unsupportive hospital system, and high workload must be addressed before implementing the model in other PHC settings.

**Supplementary Information:**

The online version contains supplementary material available at 10.1186/s12913-021-06183-z.

## Background

Diabetes is one of the most significant global health issues that affects many people worldwide [1]. The global prevalence of diabetes was estimated to be ﻿9.3% (﻿463 million people) in 2019 and predicted to rise to 10.9% (700 million people) by 2045 [2]. The Middle East and North Africa (MENA) region was ranked the second highest region globally in terms of the prevalence of diabetes, with a current prevalence rate of 12.8% [2]. Within the MENA region, the highest diabetes prevalence was found in the six Gulf Cooperation Council (GCC) countries including Qatar. In Qatar, the prevalence of diabetes was ﻿15.5% in 2019 [2], and this was predicted to rise to 29.7% by 2035 [3]. Despite the advancement in healthcare services globally, the provision of healthcare is still fragmented and complex [4] and diabetes management is still challenging [5].

The conventional care model, where physicians are the sole caregivers who address patients’ healthcare needs, is challenged to deliver holistic diabetes care [6, 7]. Several constraints limit the ability of primary care physicians to meet all the healthcare needs of the patients [8]. Indeed, physicians commonly experience high workload that often forces them to provide suboptimal care [9]. Diabetes cannot be adequately managed by a single healthcare professional (HCP) group [10]. Multidisciplinary healthcare teams involve a variety of HCPs, including nurses, physicians, pharmacists, dieticians, and others who share and combine their skills, expertise, and resources to provide comprehensive, patient-centered care to patients with diabetes instead of an episodic, fragmented, and disjointed form of care [11]. HCPs were globally recognized for their ability to manage up to 77% of chronic and preventive care in isolation from or collaboration with other HCPs, potentially offsetting some demand for physician services while improving access to care [8]. No diabetes mellitus (DM) management approach is as important as the services of a specialized diabetes healthcare team.

Collaborative Care Model (CCM) comprises multiple HCPs with different professional backgrounds working together in collaboration with patients, families, caregivers, and communities to deliver optimal care [12]. There is a substantial body of evidence highlighting the positive impact of CCM in reducing medical errors and improving patients and health outcomes [4, 13–15]. Education and self-management support provided by nurse practitioners as part of a collaborative team helped 50% of patients to achieve target glycated hemoglobin A1c (HbA1c), 95.6% to achieve a target systolic and diastolic blood pressure, and 57.8% to achieve target low-density lipoprotein cholesterol [16, 17]. Similarly, the addition of pharmacists to the healthcare team reduced HbA1c, fasting blood glucose, body mass index, body weight, blood pressure, hospitalizations, risk of diabetes-related complications, and mortality as well as improved patients’ knowledge about diabetes and its complications, its treatment, self- monitoring [18–20], and their quality of life (QoL) [21]. Although the value of each healthcare team member is well-recognized globally, multidisciplinary team collaboration in primary healthcare (PHC) settings is still underutilized due to non-referral and lack of perceived need for the service [22].

The perspectives of HCPs and patients with diabetes attending primary care have not been widely investigated. Physicians perceived interprofessional teamwork as an opportunity for delegating patient education to nurses and diabetes educators and monitoring diabetes medications to pharmacists [23]. Physicians valued the positive impact of engaging other HCPs on patient’s knowledge and other health outcomes [9, 23]. Moreover, pharmacists also expressed that working in a team that includes community health workers had produced positive clinical outcomes in patients with uncontrolled diabetes [24]. On the other hand, patients had a favourable opinion about and high satisfaction with their disease management and collaborative treatment after participating in a CCM of diabetes management [16, 25, 26].

Although some global evidence supports the positive perception of CCM offered for patients with diabetes in PHC settings, studies investigating the perspective of HCPs and patients regarding the value of CCM in diabetes management in PHC settings in the MENA region, especially in Qatar, are scarce. This study will describe the components of the CCM provided by HCPs in Qatar Petroleum Diabetes Clinic (QPDC), as well as the perceived factors that affect CCM implementation and provision, which can guide the future implementation of CCM in other ambulatory settings if proven to be effective and well-perceived. Managing and controlling diabetes will help in reducing the complications, risks of hospitalizations, and medication overuse associated with the disease, resulting in cost-savings and better resource utilization, thus reducing the burden of the disease. Therefore, the primary aim of this research study was to explore the perspectives of patients with diabetes attending PHC settings and their HCPs on the value of CCM in diabetes management.

## Methods

### Study design

An exploratory single-case research methodology has been used. This approach is acceptable in the sense of the present investigation as a case study investigates “contemporary phenomenon in its real-life context in particular where the boundary between phenomenon and context is not clearly evident” [27]. The perspectives on the value of CCM was considered as the “phenomenon” of interest, which was researched within the “defined context” of CCM for diabetes care in Qatar, in the period between 2019 and 2020, the “defined unit” [28]. The design of a qualitative study using semi-structured face-to-face interviews was considered in this study as we aimed to explore in depth the perspectives of HCPs and patients about the value of CCM in diabetes management. This method is widely used in health services research where a summary of the experience of HCPs or patients with a particular phenomenon is needed [29].

### Study setting

This study was conducted at the QPDC in Dukhan. QPDC was established in 2007 as part of Qatar Petroleum Medical Center (QPMC) in Dukhan. QPMC offers a variety of healthcare services to QP employees and their families, and other members of the community including Qataris and residents. The clinic is staffed with over 20 HCPs and serve 327 patients with diabetes. To our knowledge, QPDC is one of the few specialized clinics in Qatar where HCPs optimally offer CCM for patients with diabetes since 2007. QPDC operates on Mondays and Wednesdays from 7:30 to 14:30, with an open walk-in policy.

### Study population and eligibility criteria

Eligible HCPs were those practicing in Qatar for a minimum of 1 year, working full-time at QPDC, involved in diabetes management, and able to speak English and/or Arabic. Eligible patients were adults (≥18 years), diagnosed with type 2 diabetes, followed up at QPDC, and able to speak Arabic and/or English.

### Collaborative care model at Qatar Petroleum diabetes clinic

The multidisciplinary team at the clinic consists of physicians, nurses, and pharmacists who have adequate educational backgrounds, credentials, and professional experiences in diabetes management. The diabetes team typically provides personalized patient education, develop treatment plans and priorities, and design appropriate action plans in consultation with the patient. There were no monetary incentives given to HCPs for providing such care. However, the Healthcare Department at Qatar Petroleum Company allocates funding for all the resources needed by the healthcare team. Patients attend individualized and regular follow-up visits at least once every month. The patients are not allocated a specific time for consultation, but consultations are typically around 30–40 min compared to 15 min at other PHC centers across the country that is solely focused on physician-patient interaction [30]. During consultations, patients receive comprehensive and standardized management for diabetes, including routine assessment and treatment, drug therapy management, and early detection of microvascular and macrovascular complications through eye screening, foot examination, kidney function test, blood pressure measurement, and lipid profile measurement.

### Data collection methods (semi-structured interviews)

Face-to-face semi-structured interviews with an average duration of 30 min per interview were conducted by the first author between August and November 2019. No third-party was present besides the participants and researchers in any of the interviews. Interviews were recorded per participant’s consent. Audiotape-recorded interviews were transcribed verbatim, while unrecorded interviews were written as field notes.

### Topic guide

The interview topic guide (**Supplementary material** **1**) was developed through a comprehensive literature review [31–34] and consideration of the research objectives. The guide included open-ended questions with probes on: demographics, understanding of collaborative care definition, criteria of teamwork, managerial support, patient’s involvement in decision-making, factors affecting CCM provision, benefits/harms of team-based care, and benchmarking to other clinics.

﻿The guide was discussed and approved by the research team, peer-reviewed by three HCPs (head physician, head nurse, and head pharmacist) working at QPDC, and modified accordingly. Finally, the interview was piloted on one HCP (pharmacist) to obtain feedback on clarity, inclusiveness, and appropriateness of the questions.

### Participants and sampling

Patients were interviewed upon completing their consultations and appointment with the healthcare team, while HCPs were interviewed based on their availability between patients’ appointments. Both groups were recruited in person on the basis of their accessibility and sociodemographic variation. Fortunately, none of the invited participants denied participation in the study. Saunders *et. al,* have described several models of saturation and their principal foci in qualitative research [35]. In the current study, data saturation was the model of saturation and data collection was considered the principal foci. In data saturation model, saturation relates to the degree to which new data repeat what was expressed in previous data [35]. Therefore, participants’ interviews continued until saturation was achieved, where new data tend to be redundant of data already collected (i.e. the researcher begins to hear the same comments repeatedly) and termination of data collection process is needed. Interviews with patients took place at the counselling room, while interviews with HCPs took place at different locations within the center (e.g. physicians’ offices).

### Data analysis

Two of the researchers (SA and AA) transcribed the interview sessions of both groups conjunctively. The researchers checked each other’s transcripts and listened to all recordings in order to achieve similarity and verify the transcripts. Using both deductive and inductive thematic analysis (for predetermined themes and emergent themes, respectively) [36–38], two researchers (SA and AA) independently reviewed the textual transcripts of all interviews, including the pilot interview, several times to gain familiarity with the participants’ responses. Meaningful phrases within the textual transcripts were coded manually, using Microsoft (MS®) Word and Excel, in order to describe the content and relevance to the research questions to ensure trustworthiness of the data analysis. Another team member (SS) validated the generated codes. Once all data were coded, the researchers compared codes pattern, and grouped relevant codes into themes (defined as something that has a certain level of pattern or meaning in relation to the research questions in the data) and subthemes using MS® PowerPoint. The identified themes were reviewed and confirmed by the research team (SA, SS, and AA) and discrepancies were resolved through consensus. Finally, quotes representing the themes were selected based on agreement from all research team members. The consolidated criteria for reporting qualitative research (COREQ), a 32-item tool for comprehensive reporting of qualitative studies, was utilized to improve the quality of reporting of this research study (**Supplementary material** **2**) [39].

### Quality measures

Quality measures or trustworthiness criteria in qualitative research including credibility, dependability, confirmability, transferability, and reflexivity [40] were assured in this study. The credibility was maintained by having interviewee responses peer-reviewed at the end of the interview by the primary pharmacist at QPDC and by implementing appropriate data analysis methods. Dependability was ensured by writing a full description of the research methodology, peer review of the interviewee responses, and reservation of all research data in one place (Google drive) that will allow other researchers to replicate the work in the future. Confirmability was maintained by keeping all research activities for future examination by other researchers. Finally, transferability was achieved by including a detailed description of the research setting and participants, and credible results interpretation.

### Research team and reflexivity

Semi-structured interviews were conducted by one of the researchers (SA). SA was a master’s student in clinical pharmacy and practice at the College of Pharmacy, Qatar University at the time of the study. She had previous experiences in conducting qualitative and quantitative research projects. A previous relationship was established between some of the participants and the research team in a pilot study conducted 1 year before the present study [41]. Participants supported the researcher’s goal of attaining a graduate degree on such an important topic. The researcher has an interest in exploring interventions and services that could reduce the burden of diabetes and previous experiences with the phenomenon in question. Quotes identification and reporting were peer-reviewed several times by the MSc supervisors to ensure transparency, quality, credibility, and eliminate to any emergent bias that could be introduced by the researchers.

### Ethical considerations

The Health Department of Qatar Petroleum Company and Qatar University Institutional Review Board (QU-IRB) approved the study (approval number: QU-IRB 1112-EA/19). The consent form and the information sheet were given to participants before the interviews. Interviewees were informed about the voluntary participation and right of withdrawal. Confidentiality was maintained by giving codes to participants.

## Results

Twelve HCPs (physicians, nurses, and pharmacists) with a mean age of 47 ± 9 (range: 29 to 64) years and 8.9 ± 5 (range: 1 to 15) years of experience in diabetes care were interviewed. The group was multinational, including six Arab and six Asian HCPs. All HCPs were attending mandatory regular continuous professional development (CPD) sessions that keep them updated about the management of diabetes. Similarly, 12 patients with type 2 diabetes for an average duration of 8.7 ± 8 years and a mean age of 53 ± 8 years were interviewed. Seven participants were Asian and 5 were Arab. Half of the participants were males and employed.

**Fig. 1:**
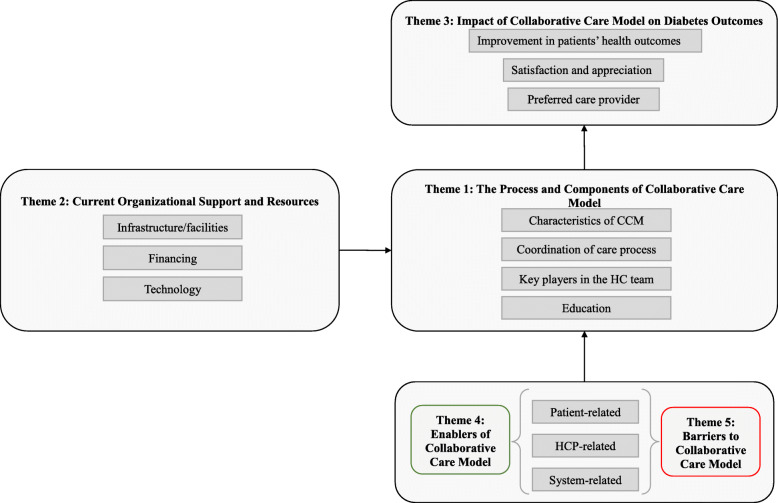
Theoretical model of CCM in diabetes management in PHC settings

HCPs’ and patients’ responses suggest that both parties have profound knowledge and appreciation of collaborative model’s positive impact on diabetes management and health outcomes. Five themes emerged from the participants’ responses including: the process and components of collaborative care model, current organizational support and resources, impact of collaborative care model on diabetes outcomes, enablers of collaborative care model, and barriers to collaborative care model (Fig. 1).

### Theme 1: the process and components of collaborative care model

#### Characteristics of CCM

Important characteristics of CCM identified include service quality and communication. Accreditation of QPDC was considered a crucial element in providing prompt and continuous provision of equitable, high-quality, non-punitive care. One participant commented: *“We are following high standards of care that is acknowledged by management. We need to stand out in care for diabetes... We reached the diamond tier, which is the highest tier in the Canadian accreditation.”* HCP9.

Further, the provision of patient-centered care requires a blame-free environment. One physician stated: *“I always tell them [patients] encouraging statements like excellent and you are doing good. I never blame the patient even if the HbA*_*1c*_ *is 12%. The next three month we will try again and again.”* HCP6.

#### Coordination of the care process

Patients have easy access to all HCPs during appointments and walk-in visits. One patient (P1) interviewed mentioned that they *“are familiar with each other... Each one of them know us very well.”* Within a single visit, *“the pharmacist checks my glucometer readings and recommends changes in medicines if needed... The nurse educates me about diet and exercise and checks my vital signs... The doctor makes tests, even if the glucose readings are regular.”* P8.

The clinic receptionists arrange frequent appointments for patients with diabetes based on their case, but rarely less than four visits a year. In the case of advanced or acute diabetes complications, *“we may refer the patient to secondary care for hospitalization in case of severe hypo-/hyperglycemia, concern about DKA [diabetes ketoacidosis] or HSS [hyperosmolar hyperglycemic state], or acute complications of diabetes.”* HCP6.

#### Key players in the healthcare team

There was a consensus among the participants, especially patients, that pharmacists and nurses were easily accessible. One patient (P5) said: *“If I need anything, I call the focal pharmacist at any time.”* Another patient Added: *“I call the diabetes nurse educator all the time when I need her.”* P10.

In order to provide valuable diabetes care, *“HCPs should have educational background, certified certificate from a well-recognized institute, experience, and CPD”* as indicated by HCP6*.* Besides credentials, the pleasant attitude of HCPs towards their colleagues and patients was highly appreciated by patients: *“Nurses and pharmacists are very good... That is why I come for diabetes education... The pharmacists are very nice, and they provide individualized follow-up... Pharmacy elsewhere only provides medicines, but here, doctors prescribe, and pharmacists help us in medications.”* P11.

Participants expressed that vigilance and proactiveness of patients are essential attributes to better health outcomes: “*… the patient has to be compliant, adherent to medications and attend follow-up appointments, read a little about his disease, find out what is his/her concerns... We provide them education, but they have to read something. The patient must have commitment to care for themselves.”* HCP6.

Active family participation in diabetes self-care in the form of group meetings with HCPs leads to better health outcomes. A HCP indicated that *“Sometimes we call for family meetings to provide special services to them like education because families need our support.”* HCP4.

#### Education and professional development

Effective diabetes management requires HCPs to remain updated with all new treatments, technologies, and modalities, *“because if you ask me about diabetes management 15 years ago it will be completely different from now... All medications, approaches, follow-up, and diagnostic criteria have changed.”* HCP 6.

Patient education is viewed as a cornerstone of diabetes management as it empowers patients to self-manage themselves, which eventually improve their diseases state. HCP4 stated: *“Patient education is the first step for us. After that, we ask the patient to bring all what he is taking from different location or from his country, even herbal products if he is taking. We try to know how he uses all these medications and then we check patient file from electronic profile.”*

### Theme 2: current organizational support and resources

#### Infrastructure

The management provided the staff with a *“private consultation room with rounded table, with suitable, comfortable chairs and education tools”* that maintains patients’ privacy and ensures their comfort throughout the duration of the visit as noted by HCP4.

#### Technology

The management at QPDC *“provide us with mobiles, technology, and free glucometers for patients”* that facilitate care provision to patients with diabetes according to HCP7*.*

#### Financing

QP Company covers all health expenses of employees. However, one participant added that for non-QP employees *“we still give them glucometer and strips, because it is difficult for them to purchase.”* HCP4.

### Theme 3: impact of collaborative care model on diabetes outcomes

#### Improvement in patients’ health outcomes

Before the launch of the service, the incidence of diabetes emergency visits due to hypo- and hyper-glycemia at QPDC *“were very high”* as indicated by HCP9*.* After the implementation of CCM, patients are having better glycemic control and hence fewer emergency visits due to *“fluctuations in their blood glucose level”* (HCP9). One patient (P1) added: *“They gave me good diet and I am almost stopping NoVo rapid”.*

A healthcare professional added: *“A lot of patients who come with HbA*_*1c*_ *12%, reach 7% and feel much better. Even their spirit, they feel like they are doing very good job. This is our job to support and promote the patient.”* HCP6.

A minority of patients expressed their concern when dealing with apathetic physicians in other PHC centers who raise their anxiety levels regarding their health status. One patient (P11) stated: *“some doctors will make you panic. The staff is very friendly because they will calm us down and reduce our panic.”*

#### Satisfaction and appreciation of CCM

Patients appreciated the useful information about health and medications provided to them by HCPs that are otherwise not provided at other PHC centers or hospitals. One participant commented: *“When we provide the service for the patient, most of them are feeling happy. When patient starts to work with us and starts to build trust between us, immediately asking why when we go to other healthcare centers or hospitals they are not telling us like that. It is the very first time for us to hear this information.”* HCP4.

The improvements in patients’ health after receiving the CCM boosted the satisfaction of HCPs as indicated by participants: *“When I get a good lab result, they become very happy and you can see that from their face.”* P1.

#### Preferred care provider

Patients showed their preference for the care received at QPDC over other clinics due to the visible health outcomes they experienced. One patient mentioned: *“I only follow-up with this clinic. Even if they are on vacation, I wait until they come back. I do not go to other clinics... I will be harmed if I follow-up in other clinics because I got used to all the HCPs here as a group.”* P5.

### Theme 4: enablers of collaborative care model

#### Patient-related facilitators

Patients indicated that having fixed healthcare staff for their case management was a factor in optimal diabetes care. Patients believed that it is their responsibility to *“create the good atmosphere for them. Because they are human beings and they are caring for us. When they spend a lot with a patient we should wait and not get angry because HCPs are not telling stories, they are giving medicines. We should have good attitude towards them.”* P4.

#### HCPs-related facilitators

In order to provide the best collaborative care to patients with diabetes, HCPs must be *“competent, well-educated, and have a desire to help the patient.”* HCP1.

Not to mention, following work ethics that are set by the health management at QP company as well as international practice guidelines can positively influence the provision of CCM. Patients recognized that HCPs who are interested in their work are more likely to be pleasant and seek patients’ preferences when making decisions: *“The doctor supervise the treatment plan, discuss, hear me, and talk friendly. The pharmacist doesn’t refuse anything I ask for, he also discusses with me in a friendly language. The nurses are patient with us and educate us.”* P5.

#### System-related facilitators

One of the repeatedly mentioned facilitators is the flexibility of the appointment system that provides HCPs with close monitoring of patients’ cases. A healthcare professional listed some of the facilitators to the service including *“flexibility in appointment system, cooperation, update staff, shared decision making with HCPs and patients, and privacy.”* HCP7.

The availability of resources, including educational materials, *“mobiles, free glucometers for patients”* (HCP7), facilitates the provision of collaborative care to improve their knowledge and ability to self-manage their condition.

### Theme 5: barriers to collaborative care model

#### Patient-related barriers

Patients who are careless about their health impose a great obstacle to the provision of CCM: *“If patients are careless, we will not be able to help them. In the meantime, it is up to the patient. We provide them with everything, but if they insist not to care and hurt themselves, it is up to them. But we still need to find out why”* (HCP6). Another barrier to care identified by HCPs is the *“lack of patient education specially geriatrics.”* HCP7.

HCPs and QPDC management address patients’ financial barriers by discussing insurance issues with patients and justifying patients’ needs to the corresponding insurance company: *“They have a limited quantity to supply to patients. If they exceed, insurance may object so they have to give an explanation.”* P1.

#### HCPs-related barriers

Some physicians at other clinical settings persistently believe that doctors should be the sole caregivers and decision-makers, which does not guarantee the provision of comprehensive care. HCP5 indicated that *“each HCP have their own mind, but here we hear from others... Even if we disagree, we try to reach one final decision at the end... Sometimes they underestimate other members of the team.”* Patients expressed frustration with the hierarchical behaviour of physicians when receiving care at other healthcare centers or hospitals. Patients disliked *“HCPs who think that they are smarter than patients, and not using good terms when communicating with patients and with each other.”* P6.

Physicians’ desire to collaborate with other HCPs is not always sufficient for providing CCM. There was a consensus that *“doctors would like to collaborate, but they have a time pressure... They don’t have the chance to work with a team... They have a lot of patient load.”* HCP1.

Having more staff possessing specialized credentials (e.g. specialized diabetes educators) was perceived as necessary for the continuity of care provision, especially *“in case the other specialized nurse is on leave”* (HCP10)*.* Moreover, participants indicated that there is a need to have specialized physicians (e.g. diabetologist, ophthalmologists) at PHC settings to eliminate the need for referring patients to secondary level of care or other hospitals.

#### System-related barriers

As strong social relationships positively impact patients’ health, the opinion of inexperienced individuals regarding health and medications are taken seriously by many patients. Patients’ obedience to such opinions interferes with professional HCPs’ advice they receive, thus leading to adverse health outcomes. A participant recognized that there is a *“social problem, especially in gulf areas, people trust what their community is telling them (e.g. not take insulin because some people took it and died or have kidney problems). So, patients trust their acquaintances blindly.”* HCP4.

Substandard and unsupportive leadership *“can discourage everyone from healthy collaboration”* as identified by P9*.* Complicated appointment systems at healthcare institutions contribute to infrequent follow-up and management of patients with diabetes. The same patient mentioned that governmental health centers have *“solved the issue by having a separate department working on arranging appoints... It is a little better now... They will give an appointment every three months and in case of urgency, you will get an appointment after a month.”*

HCPs indicated that comprehensive care provision is not possible in the case of dysfunction of electronic healthcare systems and tools (e.g. glucometer). Although QPDC provides patients with glucometers that are linked to the MIMS system, *“not having a glucometer software that connects with different types of health systems”* was perceived as a barrier to CCM provision by HCP8.

## Discussion

To our knowledge, this was the first study in Qatar to investigate the perspectives of patients with diabetes and HCPs providing care for those patients regarding the value of CCM in diabetes management in PHC settings. As one previous study suggested, the introduction of new working relationships may not always be successful due to the lack of demonstrable achievements and poor role definitions as well as poor relationships [11]. Therefore, it is logical to explore the perspective of key stakeholders regarding the introduction of CCM for diabetes in PHC settings. This study supports the development and implementation of CCM in diabetes management in PHC settings in Qatar and the greater Middle East.

Both patient and HCP participants alike had expressed the importance and value of collaboration in the healthcare environment, which ultimately reflects the harmony in the provision of care to patients with diabetes. Clinical interactions among HCPs created new knowledge and understanding of the patient, thereby enabling the provision of individualized patient care [11]. The primary reason why patients prefer CCM at QPDC over usual care at other PHC settings is the availability of unique characteristics exclusive to this clinic. Patients perceived the easy access to qualified HCPs, the easy arrangement of follow-up visits, and receiving special health education as important determinants to self-manage their condition. ﻿Participants in one study were also aware that knowledge and education alone is not sufficient to manage diabetes safely and effectively [42, 43].

Formal and informal communication was frequently valued as a crucial component of CCM as it reflects on the quality of the care delivery and patients’ familiarity with the healthcare provider. Studies have shown that familiarity and informal interactions (e.g. personal interactions during lunch, providing corridor consultations, etc.) have promoted personal exchange, encouraged intergroup ﻿relations, enhanced the understanding of each profession’s approaches, and clearly defined different professional priorities of different HCPs [44, 45]. Furthermore, HCPs recognized that face-to-face meetings facilitated the provision of better patient-centered care and facilitated timely responses during patients’ follow-up while patients were still on site [11]. Conversely, the lack of informal relationships was shown to disconnect HCPs from their healthcare team [11], and that is why interpersonal communication was referred to as the ‘glue’ of multidisciplinary collaboration [46]. HCPs shall encourage healthy discussions and respect patients’ concerns, as this would be a more useful way to correct the patients’ understanding of diabetes and gain their cooperation [9].

As patients tend to focus on their experience of illness, HCPs focus on the physiological aspects of illness and its management [47]. Although both aspects are important to patients’ health and well-being, differences in views can result in communication misalignment [47, 48]. In the present study, participants indicated that there is a need for efficient communication and education to reach a common ground. Therefore, HCPs must include patients in the decision-making process to facilitate shared goals, better adherence, and potentially better health outcomes [47]. In this qualitative study, all participants appreciated the inclusion and engagement of patients in shared decision-making in diabetes management, which made them feel that they are “treated humanely”. In several studies, patients expressed a desire to be “perceived as individuals, not illnesses” and indicated that they felt “reduced to their disease” when HCPs exclusively focus on solving medical problems [49]. HCPs in the current study felt that the provision of engaging, respectful, and humane healthcare was their responsibility.

Regarding the impact of CCM, it was recognized that there was a perceived increase in patients’ satisfaction and QoL as well as an improvement in patient health after receiving CCM. Other studies have reported similar results, but quantitatively [50–52]. For example, Mast et al reported a significant improvement in patients’ satisfaction with their current diabetes treatment, the amount of time taken to manage their diabetes, and the frequency of feeling physically ill on the ﻿QoL questionnaire administered to patients who received baseline usual care (pre-test) then received collaborative care (post-test) [50]. Another impact of CCM reported by the participants is the reduction of emergency department visits due to diabetes-complications. HCPs have observed that the initiation of the service had resulted in a reduction in emergency visits at QPMC, which was similar to the findings of a cross-sectional study conducted in the United States [51].

The outcomes of diabetes management can be influenced by several factors related to patients, HCPs, and the healthcare system. Several barriers to the provision of CCM for patients with diabetes have been identified in the literature as well. For example, HCPs who participated in one study were frustrated with patients’ poor adherence to a healthy lifestyle and medications recommended by the healthcare team, especially older and uneducated patients [9]. Similar to our findings, the participants of the former study indicated that older and uneducated patients with diabetes were not willing to follow physicians’ recommendations or attend health education [9]. Physicians and nurses in the same study also expressed a higher probability of medication misuse and non-adherence by patients who fear harm or damage to their body organs [9], a finding that was contrast to our findings, where patients showed no concern for diabetes consequences as they felt adequately educated and managed at QPDC.

One of the HCPs-related barriers recognized by patients is lack of allocating sufficient time for consultation by HCPs, resulting in a lack of adequate patient education and counseling on self-management. Other studies showed that patients also frequently complained that providers “go too quickly” [53] and do not have time to get to know them and their concerns [9, 47, 49]. Furthermore, HCPs recognized lack of trust and appreciation of other team members’ roles as a barrier to the provision of CCM in other PHC settings. This could be more prominent among new team members because their professional competency and ability had yet to be demonstrated [9, 11, 54]. However, mutual respect and understanding of others’ role were the most important factors for promoting effective service integration among all HCPs involved in caring for patients with diabetes [11]. The increase in familiarity among HCPs suppresses professional boundaries and hierarchies and encourages collaboration, thus enhance trust [11]. Some physician have related the lack of collaboration in the healthcare environment to mistrust of the competences of the other HCPs, knowing that the complexity of diabetes care necessitates a sharing of responsibility between HCPs [55].

Despite the increasing prevalence of diabetes, inadequate reimbursement of comprehensive diabetes care is frequently reported in the literature [56–58]. Most of the services needed by patients with diabetes are inadequately reimbursed, which limits HCPs ability to perform all the tasks necessary to deliver comprehensive diabetes care [56]. Even in a fee-for-service environment that rewards volume over quality, HCPs could not afford to provide collaborative diabetes care to all patients with diabetes, despite their willingness and interest to do so [47, 59]. At QPDC, reimbursement was not perceived as a concern, probably because of the adequate financial support from Qatar Petroleum. The lack of teamwork approach and shortage of human resources were recognized by HCPs as system-related barriers to CCM in PHC settings in general. Previous researchers found that physicians indicated that they had to do everything for the patients due to lack of diabetes specialist nurses, shortage in the number of dieticians and health educators, and no podiatrists for foot care [9, 59].

Dissemination of the project findings to stakeholders (health-policy makers, primary care HCPs who manage patients, and patients) with recommendations on how to improve CCM provision will draw more attention to the impact of interprofessional collaboration and education and will lead to optimal utilization of this care model. The applicability of such model in other PHC settings in Qatar, Gulf region, and the Middle East region is very feasible for several reasons. First, there is high demand for diabetic collaborative care services as the prevalence of diabetes in Qatar and the Gulf region is rising with an alarming rate. Second, the country has been studying several interventions to limit the burden of diabetes, and has emphasized on the importance of collaborative care in diabetes management in PHC settings in the National Health Strategy. Third, other PHC settings are awaiting the evidence on the effectiveness of CCM for diabetes management, and studies that showcase the factors affecting its implementation, especially barriers, that can be easily addressed. Fourth, the number of staff involved in providing CCM and their duties is explicitly described, and can be undoubtedly replicated in other PHC settings with similar patient population size and characteristics, efficient resources allocation, and administrative support.

The coalition of HCPs’ and patients’ perspectives in this project allowed the generation of detailed, powerful, and compelling results based on stakeholders’ experiences and observations, and revealed the value of CCM that cannot be investigated quantitatively. All HCP participants were familiar with the researcher and were consistent in the information they provided, obviating the concern of data manipulation by the researcher. We have strengthened the credibility of this study by interviewing participants of diverse sociodemographic characteristics to introduce variability to the study sample and answer the research questions from different perspectives. The identified facilitators and barriers to CCM provision in PHC settings for patients with diabetes may guide health policymakers to consider both factors when implementing CCM. Despite these strengths, this study has some limitations to be considered. The presence of the researcher during the interviews might have created some anxiety in some of the participants. Participants’ anxiety was addressed by allocating adequate time to establish rapport before the interview. Also, the purposive sample was determined based on HCPs’ recommendations, which could introduce bias beyond the researcher’s awareness.

## Conclusion

This study is the first to investigate and report the positive perspectives of patients and HCPs on the value of CCM for managing diabetes in primary care settings. Overall, the study has managed to contribute substantial additional information regarding the value and impact of multidisciplinary care model in diabetes within a primary care setting, highlighting demonstrable achievements, such as clear role clarifications and professional relationships between HCPs and patients, perceived positive health outcomes, and feasibility of implementing the model in other PHC settings. Important components of CCM such as HCPs’ and patients’ attributes and attitudes, family involvement in the care process, availability of technology, and utilization of facilities were recognized by the participants. The provision of CCM may promote patients’ health outcomes, level of patients and HCPs satisfaction, and preference of the model over other forms of care. To facilitate collaborative practice in similar settings, pleasant attitudes of patients and HCPs besides administrative support through tangible resources must be considered. However, undesirable attributes of HCPs and patients, unsupportive hospital system, and high workload were some of the identified barriers to CCM provision to patients with diabetes in PHC settings. Understanding of the complexity of factors that influence CCM provision for patients with diabetes in PHC settings provides HCPs and health policymakers with future directions.

## Supplementary Information

**Additional file 1.** Semi-structured Interview Guide.

**Additional file 2.** COREQ (COnsolidated criteria for REporting Qualitative research) Checklist.

## Data Availability

Raw data (i.e. interview transcripts) are available upon request.

## Abbreviations

CCM: Collaborative Care Model
COREQ: Consolidated criteria for reporting qualitative research
CPD: Continuous professional development
DM: Diabetes mellitus
GCC: Gulf Cooperation Council
HbA1c: Glycated hemoglobin A1c
HCP: Healthcare professional
MENA: Middle East and North Africa
PHC: Primary healthcare
QoL: Quality of life
QPDC: Qatar Petroleum Diabetes Clinic
QPMC: Qatar Petroleum Medical Center
QU-IRB: Qatar University Institutional Review Board
