# Supplementary material for: Collaborative care model for diabetes in primary care settings in Qatar: a qualitative exploration among healthcare professionals and patients who experienced the service
Source: BMC Health Serv Res. 2021 Mar 2;21:192. doi: 10.1186/s12913-021-06183-z (PMC7927378; doi:10.1186/s12913-021-06183-z)
Supplement: Supplementary file 1 — Additional file 1. Semi-structured Interview Guide. [file 12913_2021_6183_MOESM1_ESM.pdf]

The perspectives of healthcare professionals and patients on the value of collaborative care model for diabetes mellitus in primary care settings

Semi-structured Interview Guide – Healthcare Professionals (Dukhan)

Introduction

Hello. Thank you for coming today. I am (facilitator's name) from Qatar University. I will be conducting today's interview with you. Also, here is my colleague (notetaker's name), who will help take notes.

Today's interview is part of a study that evaluates the value of collaborative healthcare for patients with diabetes. Today, you will be asked to share your perspective on the value of collaborative diabetes care at the Diabetes Clinic of Dukhan Healthcare Center to highlight the importance of applying such care for patients with diabetes in primary settings.

Initial neutral question and demographics

Ask interviewee to introduce themselves, their age, nationality, health care profession/professional role, year of graduation (first professional degree to practice), and years in practice in diabetes management.

General introductory questions

1. When you think about health care being provided by a team, what does that mean to you?
    a. I will define collaborative healthcare if the participant fails to correctly define or describe World Health Organization definition of interprofessional collaborative practice: "Multiple health workers from different professional backgrounds working together with patients, families, caregivers and communities to deliver the highest quality of care" (WHO, 2010).
    b. Did you also come across the term "collaborative care model (CCM)"? A measurement- guided care based on evidence-based practice guidelines that focusses particular attention on patients not meeting their clinical goal (American Psychiatric Association)
2. In your opinion, what are the criteria/characteristics of collaborative care and teamwork? (Hint: E.g. Communication)

A. Description of the service at the clinic

1. Who are the key players (i.e. main members of the healthcare team involved) in providing diabetes care at the clinic?
2. How do you work with other healthcare professionals in your team to provide care for patients with diabetes? Who does what?
3. How do you work together as a team to come up with a plan for patients (i.e. process of care provision)?
4. How do the team members communicate with each other and with the patients?
5. How do you also provide collaborative care outside routine appointments? Can patients communicate with you between appointments? What is your commitment to providing patients with needed information?
6. What support do you receive from the management of the center in terms of infrastructure, facilities, and human resources?

B. Patient's involvement in decision-making

1. What is the role of patient in CCM and team-based care? Is the patient's role helpful in addressing his/her needs?
    a. IF NO: To what extent do you intend to make the patient part of the healthcare team?
    b. IF YES: What needs are being met now? How is that happening?
    c. How does engaging the patient in the healthcare team and partnership in decision-making help/discourage the patient and you?

C. Impact of CCM

1. How is team-based health care good or bad for patients' health?
    a. What might be good for patients about team-based care? How does it benefit the patients? (Hint: self-care and self-management)
    b. What, if anything, might be bad for patients about team-based care? How might it harm or fail to help patients?
2. Describe for me how you feel about the efforts you invest in providing CCM to patients with diabetes? Do you believe it is more convenient for you to deliver usual care model compared to CCM?
3. In your opinion, what are the harms of not providing CCM to patients with diabetes?
4. What is the patients' impression about CCM in Dukhan? To what extent do they appreciate the service?

D. Benchmarking

1. What characteristics are unique to the Diabetes Clinic at Dukhan Healthcare Center compared with similar clinics in other primary healthcare centers in Qatar? (Hint: environment, location, shared decision-making, etc)
2. Why other primary healthcare centers do not provide CCM?

E. Barriers and influencers

1. What factors do you believe are barriers for the provision of CCM in diabetes?
2. What factors do you believe are enablers/facilitators for the provision of CCM in diabetes?

Concluding

1. What do you suggest could improve the provision of CCM in diabetes management (if it needs improvement) or maintain it?
2. Would you like to share any additional information related to this topic?

Thank you very much for your time today and for your honest opinion and fruitful discussions. We greatly appreciate your participation in this study.

كلية الصيدلة
College of Pharmacy
QATAR UNIVERSITY جامعة قطر

The perspectives of healthcare professionals and patients on the benefits of collaborative care model for diabetes mellitus in primary care settings

Semi-structured Interview Guide – Patients (Dukhan)

Introduction

Hello. Thank you for coming today. I am (facilitator's name) from Qatar University. I will be conducting today's interview with you. Also, here is my colleague, (notetaker's name), who will help with taking notes.
Today's interview is part of a study that evaluates the value of collaborative healthcare for patients with diabetes. Today, you will be asked to share your perspective on the benefits of collaborative care at the Diabetes Clinic in Dukhan Healthcare Center to highlight the benefits of applying such care for patients with diabetes in primary healthcare settings.

Initial neutral questions and demographics

Ask interviewee to introduce themselves, their age, nationality, highest level of education, employment status, type of diabetes and for how long they had diabetes, and how long they have been in Qatar.

General introductory questions about diabetes and collaborative care

3. Please help me understand what diabetes is?

A. Defining and describing Collaborative Care Model (CCM)

1. When you think about health care being provided by a team, what does that mean to you?
    a. I will define collaborative healthcare, if the participant fails to correctly define or describe (see below).

    The World Health Organization (WHO) definition of interprofessional collaborative practice: "Multiple health workers from different professional backgrounds working together with patients, families, caregivers and communities to deliver the highest quality of care" (WHO, 2010).

2. When you think about healthcare provided by a "team" at the clinic, what indicates to you that you are being cared by a team?
    a. Who are the main members of health care team who provide you with care for diabetes in the clinic?
    b. What are the different health care services you receive from the different team members? Who does what?
    c. How does your health care team coordinate or manage your health care needs for diabetes? (process of care provision)
    d. Does the team members work together to come up with a plan for your care?
    e. How do the team members communicate with each other and with you?
3. How often do you visit the clinic for your diabetes follow-up?
4. In the last month, approximately how many different health care professionals have you seen (e.g. doctors, pharmacists, nurses, physical therapists, etc) at the clinic and elsewhere?
5. Who do you seek for advice regarding diabetes or diabetes complications between appointments?

B. Patient's involvement in decision-making

7. Do you feel that you are involved in your health care in a way that meets your needs for diabetes?
    a. IF NO: What would make it better?
    b. IF YES: What needs are being met now? How is that happening?
    c. To what extent do you feel that you are part of decision making of your health care team? Is it a good thing or a bad thing?

C. Impact of team-based care

1. How is team-based health care good or bad for patients' health?
    a. How has it benefited you? How does it improve your care and your experiences as a patient? (Hint: self-care and self-management)
    b. What, if anything, might be bad for patients about team-based care? How might it harm or fail to help you?
2. How do you feel after appointments with diabetes educators? Does the advice provided to you improve your ability to manage diabetes till the next visit?

D. Benchmarking

1. What qualities attract you to seek diabetes care at this center over other primary healthcare centers?
    a. What do you find helpful in appointments? (Hint: environment, location, shared decision-making, etc)
    b. Why do the services (if any) by other primary healthcare centers do not attract you?

E. Barriers and enablers/facilitators

1. What factors do you believe are barriers for the provision of team-based care in diabetes?
2. What factors do you believe are facilitators for the provision of team-based care in diabetes?

Concluding

3. Do you have any additional suggestions/comments/questions about team-based care for diabetes? Have we missed something you think is important?

Thank you very much for your time today and for your honest opinion and fruitful discussions. We greatly appreciate your support.
